# Association of angiotensin-converting enzyme inhibitors and angiotensin-receptor blockers with risk of mortality, severity or SARS-CoV-2 test positivity in COVID-19 patients: meta-analysis

**DOI:** 10.1038/s41598-021-84678-9

**Published:** 2021-03-03

**Authors:** Mohitosh Biswas, Most. Sumaiya Khatun Kali

**Affiliations:** Department of Pharmacy, University of Rajshahi, Rajshahi, 6205 Bangladesh

**Keywords:** Outcomes research, Viral infection

## Abstract

The effects of angiotensin-converting enzyme inhibitors (ACEIs) and angiotensin II receptor blockers (ARBs) in the treatment of COVID-19 are highly debated. This study was aimed to assess aggregated risk by investigating the association of ACEIs/ARBs users against non-users of ACEIs/ARBs with the risk of mortality or severe clinical manifestations or magnitude of SARS-CoV-2 test positivity in COVID-19 patients. Systematic literature search was carried out in different databases for eligible studies. The pooled relative risks (RRs) were measured using RevMan software where *P*<0.05 was set as statistical significance. In total, 10 studies were included in this analysis. After pooled estimation, it was demonstrated that SARS-CoV-2 positive patients taking ACEIs/ARBs were not associated with an increased risk of mortality compared to those not taking ACEIs/ARBs (RR 0.89; 95% CI 0.64–1.23; *P*=0.48). Furthermore, the risk of composite severe clinical manifestations was not significantly different between the positive patients with or without ACEIs/ARBs users (RR 1.29; 95% CI 0.81–2.04; *P*=0.28). There was no risk difference for SARS-CoV-2 test positivity in patients with or without ACEIs/ARBs users (RR 1.00; 95% CI 0.95–1.05; *P*=0.91). These findings may augment current professional society guidelines for not discontinuing ACEIs/ARBs in treating COVID-19 patients where it is clinically indicated.

## Introduction

Coronavirus disease 2019 (COVID-19) caused by severe acute respiratory syndrome coronavirus 2 (SARS-CoV-2) has led to substantial morbidity and mortality throughout the world since it was first detected in early December 2019 in Wuhan, China^1^. It has already been established that SARS-CoV-2 binds to the angiotensin-converting enzyme 2 (ACE2) receptor of the extracellular domain of the transmembrane to gain entry into host cells^1,2^. Angiotensin-converting enzyme inhibitors (ACEIs) and angiotensin II receptor blockers (ARBs) are frontline therapies in treating cardiovascular disorders including hypertension and diabetes; however, they may upregulate ACE2 expression in some animal models^1,3,4^. Data remains limited for human studies showing variations on plasma ACE2 levels, most importantly no study has identified their effect on lung-specific expression of ACE2^1,5^.

It is alarming that COVID-19 patients with comorbidities especially hypertension, diabetes, and cardiovascular diseases, have been found to be associated with the highest adverse clinical outcomes, e.g. deaths^6,7^. This may lead to clinical concerns that patients who are taking ACEIs/ARBs for combating cardiovascular diseases along with hypertension and diabetes are at an increased risk for becoming infected with SARS-CoV-2 and are at the highest risk of worst clinical outcomes^1,8^. However, in this clinical debating situation, it has also been postulated that ACE2 upregulation may improve clinical outcomes in SARS-CoV- 2 infected patients by protecting against lung injury^9^. Furthermore, in certain high-risk SARS-CoV-2 infected patients, the removal of ACEIs or ARBs may be detrimental^5^. Despite the lack of sufficient robust evidence, several professional societies have recommended continuing the administration of these medications in patients with COVID19^1^.

It is therefore expected that researchers may have explored the associations of taking ACEIs/ ARBs with an increased risk of mortality or severe clinical manifestations (e.g. admitted to the intensive care unit (ICU)/using mechanical ventilation/deaths) or even the magnitude of positivity with the SARS-CoV-2 infection test during the COVID-19 pandemic.

To date, only one meta-analysis has appeared in the literature but has several methodological limitations^10^. First, it did not clearly group patients with or without ACEIs/ARBs users for estimating its effects on SARS-CoV-2 infection. Second, when estimating aggregated risk of clinical outcomes, e.g. mortality or severity, this meta-analysis used data from some studies that were not either found in the literature or the mother studies itself did not have clear data on the patients using ACEIs/ARBs versus no-users of ACEIs/ARBs but adjusted with covariates, which makes come controversy regarding the outcomes of this meta-analysis.

Another systematic review conducted by Mackey K et al. 2020 reported the association of ACEIs/ARBs users versus non-ACEIs/ARBs users with SARS-CoV-2 test positivity from limited perspectives^11^. However, the severity of COVID-19 illness was not reported for the patients with or without ACEIs/ARBs users but was adjusted with other covariates in this analysis, which certainly renders lacking robust evidence regarding these conflicts. Most importantly, this analysis used the majority of the data from preprint articles which is not peer-reviewed making this analysis some concerns^11^.

It is evident from the perspectives of the current controversy that a robust study is needed showing the association of clinical manifestations of COVID-19 with or without ACEIs/ARBs users. Meanwhile, new studies may appear in the literature that need to be incorporated to update the literature. This study was therefore aimed to assess aggregated risk of mortality or severity of illness or SARS-CoV-2 test positivity in COVID-19 patients with or without ACEIs/ARBs users.

## Results

### General characteristics and quality of included studies

**Figure 1:**
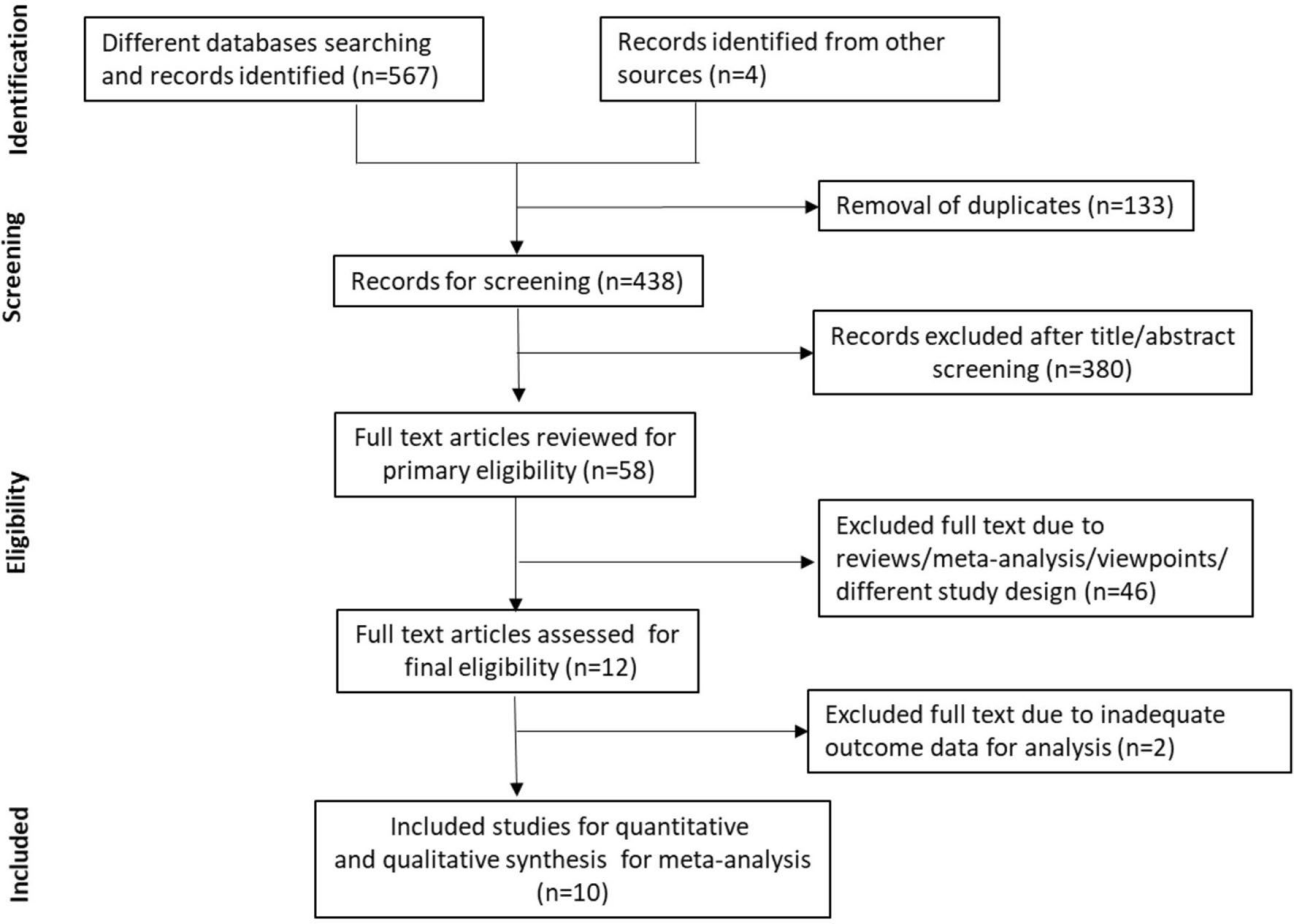
PRISMA flowchart showing the selection of studies for meta-analysis.

The selection process of included studies as undertaken following PRISMA guidelines for this meta-analysis is shown in Fig. 1. In total, 10 studies comprising 49,188 patients were included in this analysis. Of these, 6,348 SARS-CoV-2 positive patients were taking ACEIs/ARBs (only six studies separated for using either ACEIs or ARBs where 2930 patients were taking ACEIs and 2557 patients were taking ARBs) whereas 23,407 positive patients were non users of ACEIs/ARBs^1,12–20^. It is pertinent to mention here that using combination of ACEIs and ARBs in COVID-19 patients not only lower blood pressure but also may maximize organ protection and was therefore used concurrently. Also, by stabilizing and internalization of ACE2–AT1R interaction, ACEIs/ARBs could prevent COVID-19 viral entry^21^. This may be another reason for using this combination in the management of COVID-19 patients.

Out of 10, eight studies provided data on mortality outcome, seven studies provided data on composite severe clinical manifestations outcome and only two studies provided data on SARS-CoV-2 test positivity as shown in Table 1. Clinical outcomes were reported for the majority of COVID-19 patients with hypertension^12,13,15–17,19,20^ although only three studies^1,14,18^ reported outcomes with multiple comorbidities including hypertension and one study separately reported outcomes for hypertensive COVID-19 patients as well as patients with other comorbidities^16^. Out of 49,188 patients, 52.9% were male COVID-19 patients with an average age of 61.1 (IQR 50.8–70.1). Besides using ACEIs/ARBs, patients were taking other classes of drugs e.g. statins, antiplatelets, beta-blockers, antidiabetics, antidiuretics etc. Importantly, 35% COVID-19 patients had hypertension and other important comorbidities were diabetes, cardiovascular diseases, respiratory disease, kidney and liver disease, cancer etc. The important baseline characteristics of the included studies are summarized in Table 1. The quality of included studies as determined by the Newcastle–Ottawa assessment scale was of high quality (score ranges between 7 and 9), as shown in Supplementary Table 1.

**Table 1:** Baseline characteristics of included studies.

Author, Year	Ethnicity	Study design	Characteristics of Patients reporting clinical outcomes	Median age (IQR);% of male	Sample size	Reported clinical outcomes	Study duration with follow-up, days
Feng et al.^12^	China	Retrospective, multi-center case–control study	COVID-19 with hypertension	53 (40–64); 56.9	476	severe clinical manifestations	44
Li et al.^13^	China	Retrospective, single-center case series	COVID-19 with hypertension	55.5 (38–67); 52.2	1178	Mortality, severe clinical manifestations	61
Mehra et al.^14^	Asia, Europe, and North America	Retrospective, multi-center case–control study	COVID-19 with multi-comorbidities	49 ± 16*; 60	8910	Mortality	40
Mehta et al.^1^	USA	Retrospective cohort study	COVID-19 with multi-comorbidities	48 (21)*; 40	18,472	Mortality, severe clinical manifestations, SARS-CoV-2 test positivity	36
Meng et al.^15^	China	Retrospective, single-center cohort study	COVID-19 with hypertension	64.5 (55.8–69); 57.1	417	Mortality, severe clinical manifestations	44
Reynolds et al.^16^	USA	Population-based observational study	COVID-19 with hypertension and other comorbidities	64 (54–75); 50.8	12,594	Severe clinical manifestations, SARS-CoV-2 test positivity	46
Richardson et al.^17^	USA	Prospective case series	COVID-19 with hypertension	63 (52–75); 60.3	5700	Mortality, severe clinical manifestations	35
Guo et al. [18]	China	Retrospective observational study	COVID-19 with multi-comorbidities	58.5 (14.7)*; 48.7	187	Mortality	32
Yang et al.^19^	China	Retrospective, single-center cohort study	COVID-19 with hypertension	66 (61–73); 49.2	126	Mortality, severe clinical manifestations	49
Zhang et al.^20^	China	Retrospective, multi-center cohort study	COVID-19 with hypertension	64 (55–68); 53.5	1128	Mortality	52

*Age reported as mean with standard deviation (SD); IQR = Interquartile range; COVID-19 = Corona virus disease-2019; SARS-CoV-2 = Severe acute respiratory syndrome corona virus-2.

### Association of ACEIs/ARBs users vs non-users of ACEIs/ARBs with the risk of mortality in patients with COVID-19

**Figure 2:**
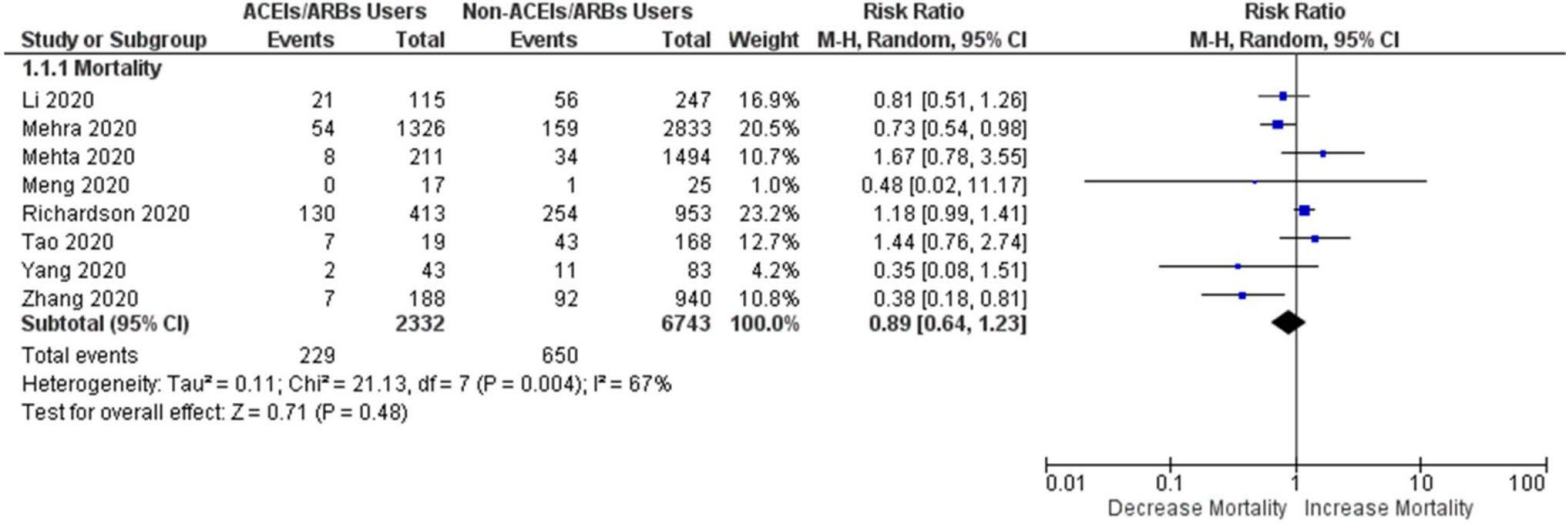
Forest plot of ACEIs/ARBs users against non-users of ACEIs/ARBs on the risk of mortality in patients with COVID-19.

The association of mortality was assessed in COVID-19 patients where 2,332 patients were taking ACEIs/ARBs and 6,743 patients were non-users of ACEIs/ARBs. As shown in Fig. 2, the results of this meta-analysis after pooled estimation indicated that SARS-CoV-2 positive patients taking ACEIs/ARBs were not associated with an increased risk of mortality compared to those positive patients not taking ACEIs/ARBs (RR 0.89; 95% CI 0.64–1.23; *P*=0.48). This finding may be considered insightful since no study has measured the pooled effects of mortality for ACEIs/ARBs users against non-users of ACEIs/ARBs in COVID-19 patients using such a large sample size. Whilst controversial whether ACEIs/ARBs should or should not continue in these patients, this aggregated finding may be considered as the highest evidence and may reduce debating in this regard.

### Association of ACEIs/ARBs users versus non-users of ACEIs/ARBs with the risk of composite severe clinical manifestations in patients with COVID-19

**Figure 3:**
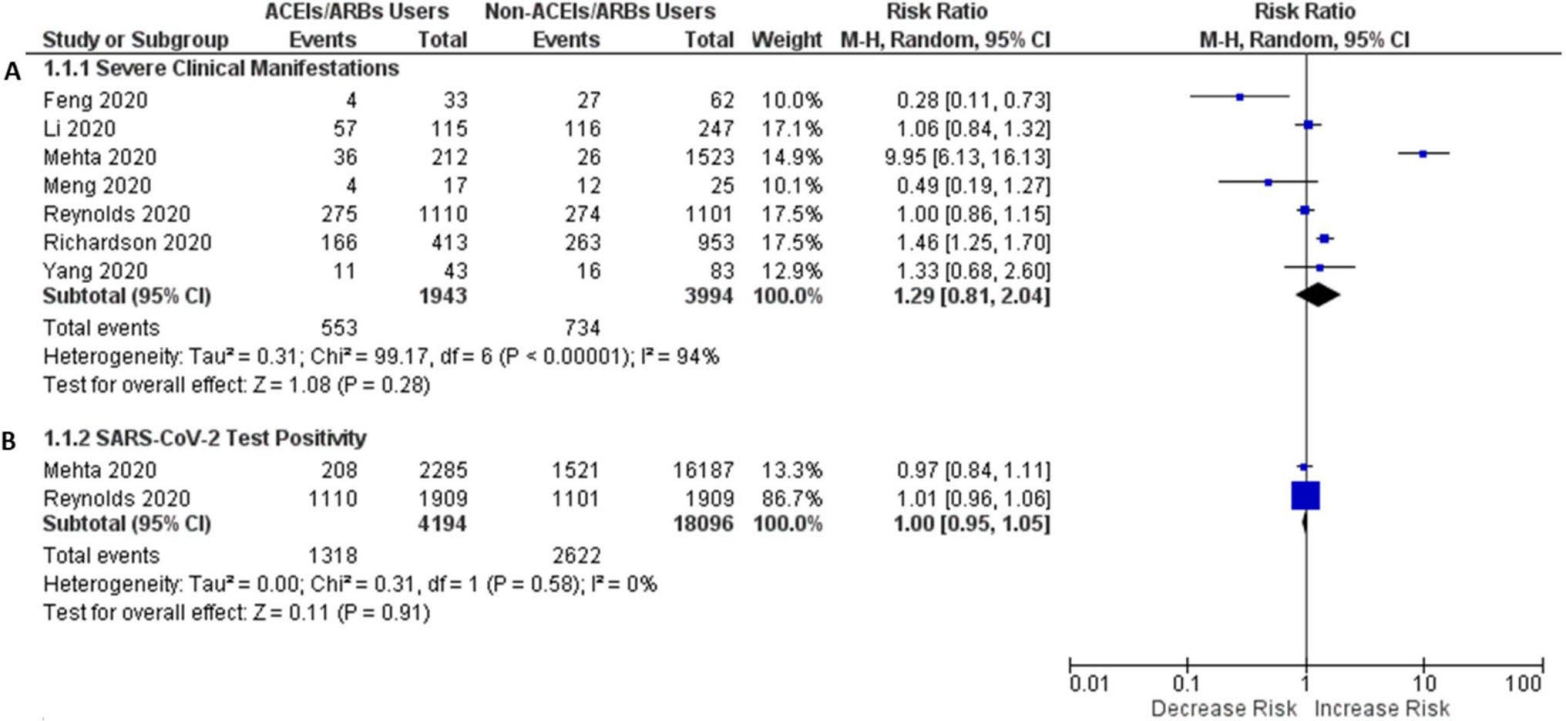
Forest plot of ACEIs/ARBs users against non-users of ACEIs/ARBs on the risk of severe clinical manifestations (**A**) and SARS-CoV-2 test positivity (**B**) in patients with COVID-19.

The risk of composite severe clinical manifestations (admitted to the ICU/using mechanical ventilation/mortality) was not significantly different between SARS-CoV-2 positive patients using ACEIs/ARBs versus non-users of ACEIs/ARBs (RR 1.29; 95% CI 0.81–2.04; *P*=0.28), as shown in Fig. 3A.

### Association of ACEIs/ARBs users versus non-users of ACEIs/ARBs with the risk of SARS-CoV-2 test positivity in patients with COVID-19

Many COVID-19 patients had multiple comorbidities and were using ACEIs/ARBs before SARS-CoV-2 testing, and many were started to use these medications after hospital admission. On the other hand, many patients were not using ACEIs/ARBs before or during hospitalization. It was of utmost importance to investigate whether prior or during hospitalization use of ACEIs/ARBs increased the magnitude of infections compared to those patients never using these medications. After pooled estimation, it was found that the magnitude of SARS-CoV-2 test positivity had no risk difference between the COVID-19 patients with or without prior or during hospitalization use of ACEIs/ARBs (RR 1.00; 95% CI 0.95–1.05; *P*=0.91), as shown in Fig. 3B. Interestingly, 10% of the COVID-19 patients using ACEIs were found SARS-CoV-2 test positive and 8.4% of the COVID-19 patients using ARBs were found SARS-CoV-2 test positive. This finding may also be considered important insights since using ACEIs/ARBs was not associated with an increased risk of SARS-CoV-2 test positivity in COVID-19 patients and it was statistically insignificant.

### Heterogeneity, sensitivity analysis and publication bias

There was significant heterogeneity in studies that tested mortality and severe clinical manifestations for ACEIs/ARBs users compared to non-users of ACEIs/ARBs. Ethnicity, age, sex and comorbidities may be accountable for a high level of heterogeneity since these variables were not adjusted for clinical outcomes in the majority of the included studies. However, heterogeneity completely disappeared in studies that assessed SARS-CoV-2 test positivity for ACEIs/ARBs users against non-users of ACEIs/ARBs. In the sensitivity analysis, no single study was found to excessively affecting the pooled effects or heterogeneity.

**Figure 4:**
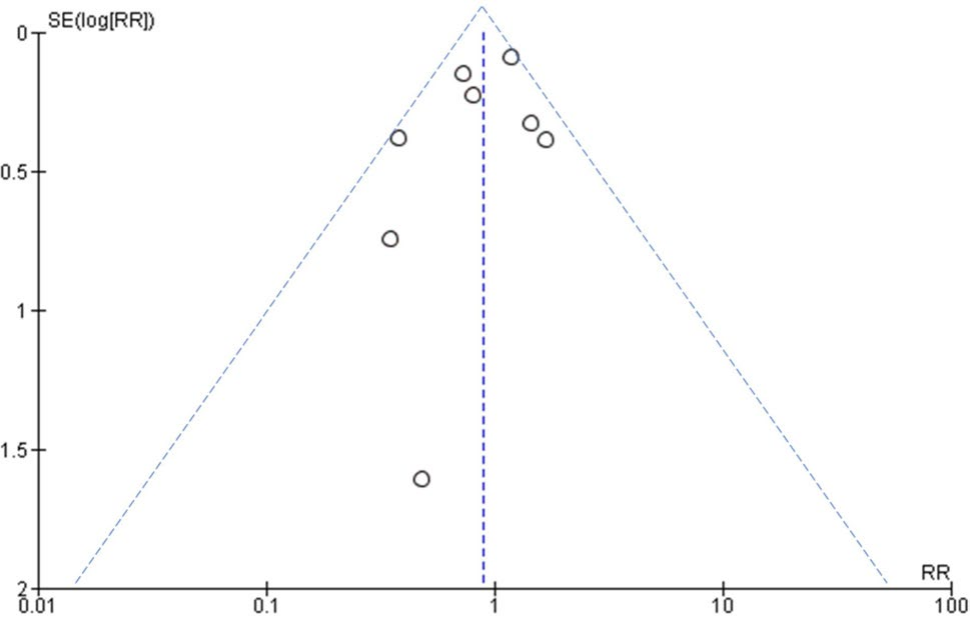
Funnel plot of ACEIs/ARBs users against non-users of ACEIs/ARBs on the risk of mortality in patients with COVID-19.

In the sensitivity analysis, it was also found that there was no risk difference for severe clinical manifestations in COVID-19 hypertensive patients with or without ACEIs/ARBs users and  it was statistically insignificant (RR 1.00; 95% CI 0.75–1.33; *P*=0.99), figure not shown here. Although the number of studies included for assessing publication bias in this analysis was very small (n = 8), even after visual inspection of the funnel plot, there was no publication bias, as shown in Fig. 4.

In summary, using ACEIs/ARBs in SARS-CoV-2 positive patients neither significantly increased the risk of viral infectivity nor severe clinical manifestations compared to those patients not using these medications. Instead, using ACEIs/ARBs in these patients was associated with a trend towards a reduction in the risk of mortality compared to those not using these medications, although results were not adjusted for other confounding factors e.g. age, sex, comorbidities etc.

## Discussion

After estimating aggregated risk, this analysis provided evidence that neither severe clinical manifestations nor magnitude of SARS-CoV-2 infectivity were significantly different with or without ACEIs/ARBs users in patients with COVID-19. Interestingly, mortality was slightly reduced (11%) in ACEIs/ARBs users of SARS-CoV-2 positive patients compared to those not using these medications although it was statistically insignificant. These findings are consistent with other analyses carried out by independent researchers^10,11^. Most importantly, the findings of the current analysis support recommendations from different professional societies for continuing ACEIs/ARBs irrespective of whether it is contraindicated for other clinical conditions, not just COVID-19 reason^1^.

Without elucidating the exact molecular mechanism, it would not be plausible to accurately explain governing the trends towards a slight reduction of mortality for using ACEIs/ARBs in patients with COVID-19 compared to those not with these medications. However, with accumulating existing evidence and data, it is reasonably believed that upregulation of ACE2 may act as a biological sword and is organ protective, e.g. protecting lung injury, myocardium and kidney, which is mainly executed by using ACEIs/ARBs in these patients^5^. From the findings of the current study, it may be postulated that the organo-protective nature of upregulated ACE2 directly induced by the ACEIs/ARBs in COVID-19 patients was probably associated with the reduction of deaths compared to those not with ACEIs/ARBs. This is because, COVID-19 patients appeared to have elevated levels of plasma angiotensin II, which in turn caused lung injury^5,22^. It was also found that COVID-19 was particularly severe in patients with cardiovascular diseases, and many of these patients had active myocardial injury, myocardial stress and cardiomyopathy provoking the severity of illness and even death^5,23–28^. This may be due to the downregulation of ACE2 for SARS-CoV-2 infection and replication which in turn may be unable to exert protective effects in organs^5,9^. The ACEIs or ARBs may particularly use in COVID-19 hypertensive patients to manage hypertension and other cardiovascular diseases where it is assumed that using ACEIs/ARBs in COVID-19 patients may upregulate ACE2 enzyme which in turn may protect organ damage and may reduce associated death as well. Since no study has proven experimentally the association between ACEIs/ARBs use and reduction of mortality in COVID-19 patients found in this study, future experimental studies are warranted to firmly establish the exact molecular mechanism behind this scientifically novel outcome.

Although using ACEIs/ARBs in COVID-19 patients in general slightly increased the risk of composite severe clinical manifestations (admitted to the ICU/using mechanical ventilation/mortality) but it failed to reach statistical significance. Importantly, when the association was assessed only for hypertensive COVID-19 patients, the risk drastically decreased, and there was almost no risk difference with or without ACEIs/ARBs users. This indicated that using ACEIs/ARBs in hypertensive COVID-19 patients actually had no significant risk differences compared to those not using these medications. A recent study^29^ conducted by Mancia et al. 2020 after multivariable adjustment indicated that neither ACEIs nor ARBs had a significant association with the risk of COVID-19, which is consistent with the findings of the present study.

The magnitude of infection positivity caused by SARS-CoV-2 was not found to be significantly different with or without ACEIs/ARBs users, which is scientifically exciting and may be considered new insights for COVID-19 patients. Again, future experimental studies are warranted to elucidate the molecular mechanism for these associations.

This is an important concern whether it should or should not continue ACEIs/ARBs in COVID-19 patients especially with hypertension because these medications are the most prevalent antihypertensive medications among all therapeutic classes used generally^17^. Because the results of this meta-analysis pooled data from individual studies that were unadjusted for known confounders, e.g. age, sex, race, ethnicity and comorbidities such as diabetes, chronic kidney disease and heart failure, the findings of the current study cannot completely address the complexity of this question. However, a recent meta-analysis indicated that COVID-19 male patients and those with age over 50 years were associated with a significantly increased risk of mortality. Comorbidities were also associated with a significantly increased risk of mortality in patients with COVID-19^6^. Although data about the impacts of ethnicity on the risk of COVID-19 in published literature remains limited, however, a systematic review conducted by Pan D et al. 2020 suggested that Black, Asian and Minority Ethnic (BAME) individuals were at an increased risk of SARS-CoV-2 infection and worse clinical outcomes from COVID-19 compared to White individuals^30^. Future clinical studies are warranted again to address these issues. However, with aggregating existing data in the literature to date, this study provides evidence that using ACEIs/ARBs in COVID-19 patients in general and in particular hypertensive patients were not associated with an increased risk for composite severe clinical manifestations or SARS-CoV-2 test positivity compared to those patients not taking these medications. Instead, using ACEIs/ARBs in these patients compared to non-using patients was associated with a statistically insignificant slight reduction in the risk of mortality. Although confounding factors e.g. age, sex, ethnicities, comorbidities were not adjusted for the current analysis, however, this evidence may be considered insightful findings in the current perspectives of controversy and lack of sufficient robust evidence in the literature.

Although this study has great findings in terms of providing evidence regarding whether ACEIs/ARBs should or should not continue with COVID-19 patients, there are some limitations of this analysis. First, it was not possible to extract data to examine the effects of age, sex, and comorbidities in ACEIs/ARBs users against non-users of ACEIs/ARBs, which may be accountable for the high level of heterogeneity. Second, due to the unavailability of data, it was not possible to estimate the dosage regimen effects of ACEIs/ARBs. Third, there was no information about the types of drugs used in users vs. non-users of ACEIs/ARBs in the majority of the included studies since there are many drugs falling into the classes of ACEIs/ARBs, which may increase the inconsistency of the outcomes. Fourth, since definition of ICU admission/mechanical ventilation/death collectively referred to as ‘composite severe clinical manifestations’ were varied slightly in the included studies, hence the risk of composite severe clinical manifestations may vary accordingly. It is therefore suggested to address these issues in future clinical studies.

In conclusions, the findings of this analysis indicated that using ACEIs/ARBs in SARS-CoV-2 positive patients was not associated with an increased risk of mortality, rather, it had a trend towards a slight reduction in the risk of mortality, although results were not adjusted for other confounding factors, e.g. age, sex, comorbidities etc. Furthermore, it was also found that neither severe clinical manifestations nor magnitude of SARS-CoV-2 infectivity were significantly different with or without ACEIs/ARBs users. These findings may augment current professional society guidelines for not discontinuing ACEIs/ARBs in treating COVID-19 patients where it is clinically indicated.

## Methods

### Literature search

The Literature was searched in PubMed from inception to May 31, 2020 following the Preferred Reporting Items for Systematic Reviews and Meta-Analyses (PRISMA) guidelines as described elsewhere^31^. Keywords were searched as "2019 novel coronavirus with ACEIs/ARBs" or "2019-nCoV with ACEIs/ARBs" or "SARS-CoV-2 with ACEIs/ARBs" or "COVID-19 with ACEIs/ARBs" or "angiotensin-converting enzyme inhibitors (ACEIs) with COVID-19/SARS-CoV-2" or "angiotensin II receptor blockers (ARBs) with COVID-19/SARS-CoV-2" or "SARS-CoV-2 with ACEIs/ARBs" AND "clinical outcomes or death or severe illness or severe clinical outcomes or test positivity with SARS-CoV-2 or clinical features/characteristics". Additionally, different important journal websites e.g. New England Journal of Medicine, Lancet, JAMA and Nature were checked for the relevant articles.

### Inclusion criteria

The studies were included if they fulfilled the following criteria: (1) studies must have two arms of COVID-19 patients in which one arm used ACEIs/ARBs whereas the other arm did not use ACEIs/ARBs; (2) studies must have reported outcomes for at least one of the three clinical outcomes in both arms; (a) mortality (b) composite severe clinical manifestations which include a combination of patients admitted to the ICU/using mechanical ventilation/mortality (c) magnitude of SARS-CoV-2 test positivity; and (3) studies must be original peer-reviewed published research that had sufficient data to be included as described above.

### Exclusion criteria

The studies were excluded based on the following criteria: (1) if the study had only one arm of treatment group using either ACEIs or ARBs but did not have another arm; (2) if the study did not clearly report outcome data for two selected arms of patients as described above; and (3) if the studies were reviews, systematic reviews, viewpoints, perspectives or correspondences.

### Data extraction, validity and quality assessment

Rayyan QCRI, a systematic review software tool^32^ was used for primary selection of the studies after importing all the literature search histories in this software following inclusion and exclusion criteria. For final selection of the studies, full texts of all primary included studies were retrieved and checked one by one. When the articles were finally selected, the full text of the included studies was carefully checked for data synthesis and quality assessment purposes. All the selection process was carried out by two investigators independently, and at the end of selection, any disagreement was resolved by principal investigator. Data synthesis and entry were double checked and validated by a third independent reviewer. To determine the quality of the included studies, Newcastle Ottawa assessment scale guidelines were followed as described elsewhere^33^.

### Statistical analysis

Estimation of pooled effects was calculated as risk ratios (RRs) and 95% Confidence Intervals (CIs) using the Mantel–Haenssel (M-H) method following either a random or fixed effect model based on the levels of heterogeneity of the included studies. Heterogeneity in the forest plot was evaluated by using the Cochrane chi-square-based Q-test and regarded as significant if the p value was less than 0.1^34^. Meanwhile, the I^2^ statistic was used to efficiently test for heterogeneity, where I^2^ < 25%, I^2^ = 25–50% and I^2^ > 50% indicate low, moderate and high degrees of heterogeneity, respectively^35^. A random effects model was used to estimate pooled effects if I^2^ > 50 and a fixed effects model was applied to calculate pooled effects when I^2^ < 50. Sensitivity analyses were carried out to measure any significant risk differences between the studies, especially if heterogeneity was found. This was accomplished by removing the included study one after another. In addition, publication bias was carried out by visual inspection of the funnel plot where symmetrical distribution of the plot indicated the absence of publication bias^36^. Funnel plot asymmetry was also assessed with Egger’s test^37^. Review-Manager software (RevMan version 5.3 Windows; The Cochrane Collaboration, Oxford, UK) was used for analyzing all data where the level of statistical significance was set as <0.05 (2-sided).

## Supplementary Information

Supplementary Information

## Data Availability

This manuscript does not contain any associated data. However, all raw and processed data used in this analysis are freely available upon request.
