# Supplementary material for: Association of angiotensin-converting enzyme inhibitors and angiotensin-receptor blockers with risk of mortality, severity or SARS-CoV-2 test positivity in COVID-19 patients: meta-analysis
Source: Sci Rep. 2021 Mar 3;11:5012. doi: 10.1038/s41598-021-84678-9 (PMC7930241; doi:10.1038/s41598-021-84678-9)
Supplement: Supplementary file 1 — Supplementary Information [file 41598_2021_84678_MOESM1_ESM.pdf]

Association of angiotensin-converting enzyme inhibitors and angiotensin-receptor blockers with risk of mortality, severity or SARS-CoV-2 test positivity in COVID-19 patients: Meta-analysis

Mohitosh Biswas, Most. Sumaiya Khatun Kali

Supplementary Table 1. Quality assessment of the included observational studies by NOS

Author	Year	Selection					Comparability			Outcome			Total Score
		1	2	3	4		5A	5B		6	7	8	
		Exposed cohort truly/somewhat representative	Nonexposed cohort drawn from the same community	Ascertainment of exposure	Outcome of interest not present at start		Cohorts adjusted for age	Cohorts adjusted for other important factor(s)		Quality of outcome assessment	Follow-up long enough for outcomes to occur	Adequacy of follow-up of cohorts	
Feng et al	2020	*	*	*	*					*	*	*	7
Li et al	2020	*	*	*	*					*	*	*	7
Mehra et al	2020	*	*	*	*		*	*		*	*	*	9
Mehta et al	2020	*	*	*	*					*	*	*	7
Meng et al	2020	*	*	*	*					*	*	*	7
Reynolds et al	2020	*	*	*	*					*	*	*	7
Richardson et al	2020	*	*	*	*					*	*	*	7
Tao et al	2020	*	*	*	*					*	*	*	7
Yang et al	2020	*	*	*	*					*	*	*	7
Zhang et al	2020	*	*	*	*		*	*		*	*	*	9

NOS=Newcastle Ottawa Scale; in this scale score ranges between 0-9 where “0-3” indicates low quality, “4-5” indicates moderate quality and score of ≥6 indicates high quality study
